# COL11A1 confers chemoresistance on ovarian cancer cells through the activation of Akt/c/EBPβ pathway and PDK1 stabilization

**DOI:** 10.18632/oncotarget.4250

**Published:** 2015-06-10

**Authors:** Yi-Hui Wu, Tzu-Hao Chang, Yu-Fang Huang, Chien-Chin Chen, Cheng-Yang Chou

**Affiliations:** ^1^ Department of Obstetrics and Gynaecology, College of Medicine, National Cheng Kung University and Hospital, Tainan, Taiwan; ^2^ Graduate Institute of Biomedical Informatics, Taipei Medical University, Taipei, Taiwan; ^3^ Department of Pathology, Chia-Yi Christian Hospital, Chia-Yi, Taiwan; ^4^ Department of Cosmetic Science, Chia Nan University of Pharmacy and Science, Tainan, Taiwan

**Keywords:** epithelial ovarian carcinoma, chemoresistance, collagen type XI alpha 1, Akt, PDK1

## Abstract

Chemoresistance to anticancer drugs substantially reduces survival in epithelial ovarian carcinoma (EOC). Here, microarray analysis showed that collagen type XI alpha 1 (*COL11A1*) is a chemotherapy response-associated gene. Chemoresistant cells expressed higher COL11A1 and c/EBPβ than chemosensitive cells. COL11A1 or c/EBPβ downregulation suppressed chemoresistance, whereas COL11A1 overexpression attenuated sensitivity to cisplatin and paclitaxel. The c/EBPβ binding site in the *COL11A1* promoter was identified as the major determinant of cisplatin- and paclitaxel-induced COL11A1 expression. Immunoprecipitation and immunofluorescence showed that in resistant cells, Akt and PDK1 were highly expressed and that anticancer drugs enhanced binding activity between COL11A1 and PDK1 binding and attenuated PDK1 ubiquitination and degradation. Conversely, chemosensitive cells showed decreased activity of COL11A1 binding to PDK1 and increased PDK1 ubiquitination, which were reversed by COL11A1 overexpression. Analysis of 104 EOC patients showed that high COL11A1 mRNA levels are significantly associated with poor chemoresponse and clinical outcome.

## INTRODUCTION

Epithelial ovarian carcinoma (EOC) is the most lethal gynaecologic malignancy [1]. Despite cytoreductive surgery followed by combination cisplatin and paclitaxel chemotherapy, many EOC patients eventually relapse, develop chemoresistant tumours, and die from the disease [2]. Therefore, it is important to identify markers that predict responsiveness to chemotherapy, which may allow for the development of therapeutic biomarkers aimed at overcoming chemoresistance.

Several studies have used gene microarrays to identify distinct genes expressed in chemoresistant EOC patients [3–6]. In this study, we analysed the expression profiles of 60 epithelial ovarian cancer tissue samples obtained at first cytoreductive surgery to identify genes associated with response to chemotherapy. Among the 30 upregulated genes selected (Table 1), collagen type XI alpha 1 (*COL11A1*) was the most highly elevated. COL11A1 belongs to the collagen family, which is a major component of the interstitial extracellular matrix. COL11A1 overexpression has been shown to be associated with progression in several cancers, including ovarian cancer [7–11]. Our previous findings also identified *COL11A1* as a disease progression-associated gene that is linked to EOC recurrence and poor survival [7]. In addition, COL11A1 is preferentially elevated in cisplatin-resistant ovarian cancer cells [12, 13]. However, the mechanisms by which COL11A1 increases chemoresistance are poorly understood. In this study, the molecular mechanisms underlying COL11A1-increased cancer drug resistance were elucidated, providing an understanding of the mode of action.

**Table 1:** The 30 genes upregulated and the 17 genes downregulated in response to ovarian cancer chemotherapy

Gene symbol	Probe ID	Log 2 ratio	*P* value	Gene symbol	Probe ID	Log 2 ratio	*P* value
POSTN	210809_s_at	3.20	0.001	MSX1	205932_s_at	−2.02	0.005
COL11A1	37892_at	2.98	0.007	SCGB2A2	206378_at	−1.94	0.001
SFRP2	223122_s_at	2.73	0.004	RNF183	235153_at	−1.93	<0.001
LUM	201744_s_at	2.70	<0.001	TMEM37	1554485_s_at	−1.65	0.006
COL8A1	226237_at	2.52	0.0020	REPS2	227425_at	−1.52	0.002
INHBA	227140_at	2.49	0.005	SPDEF	214404_x_at	−1.30	0.003
TWIST1	213943_at	1.86	0.007	KLHDC7A	1556012_at	−1.20	0.004
BEX2	224367_at	1.84	0.006	LOC100653206	230991_at	−1.19	0.008
THBS2	203083_at	1.83	0.004	STS	203767_s_at	−1.16	0.009
DCN	201893_x_at	1.78	0.007	ADCY1	213245_at	−1.13	0.004
RGS2	202388_at	1.77	0.002	SRD5A3	222750_s_at	−1.11	0.008
ISLR	207191_s_at	1.67	0.003	NRTN	210683_at	−1.10	0.003
DKK2	219908_at	1.56	0.003	COL4A4	229779_at	−1.10	0.009
COL5A2	221729_at	1.47	0.006	MESP1	224476_s_at	−1.06	0.002
CNTN1	227209_at	1.42	0.007	GALNT16	230418_s_at	−1.06	0.003
AEBP1	201792_at	1.40	0.004	CISH	223377_x_at	−1.03	0.008
WISP1	229802_at	1.39	0.008	TSPAN33	225775_at	−1.02	0.007
COL5A1	203325_s_at	1.36	0.009				
COL6A3	201438_at	1.34	0.001				
CH25H	206932_at	1.26	0.009				
FBN1	202766_s_at	1.24	0.001				
SYT11	209198_s_at	1.22	0.009				
NUAK1	204589_at	1.19	0.002				
COL3A1	215076_s_at	1.18	0.005				
FKBP10	219249_s_at	1.17	0.001				
EDNRA	204464_s_at	1.13	0.006				
LOC440434	214107_x_at	1.09	<0.001				
ZHX2	203556_at	1.02	0.010				
COL16A1	204345_at	1.00	0.005				
RSF1	222540_s_at	1.00	0.005				

## RESULTS

### Detection of elevated COL11A1 levels in chemoresistant ovarian cancer by expression profiling

**Figure 1:**
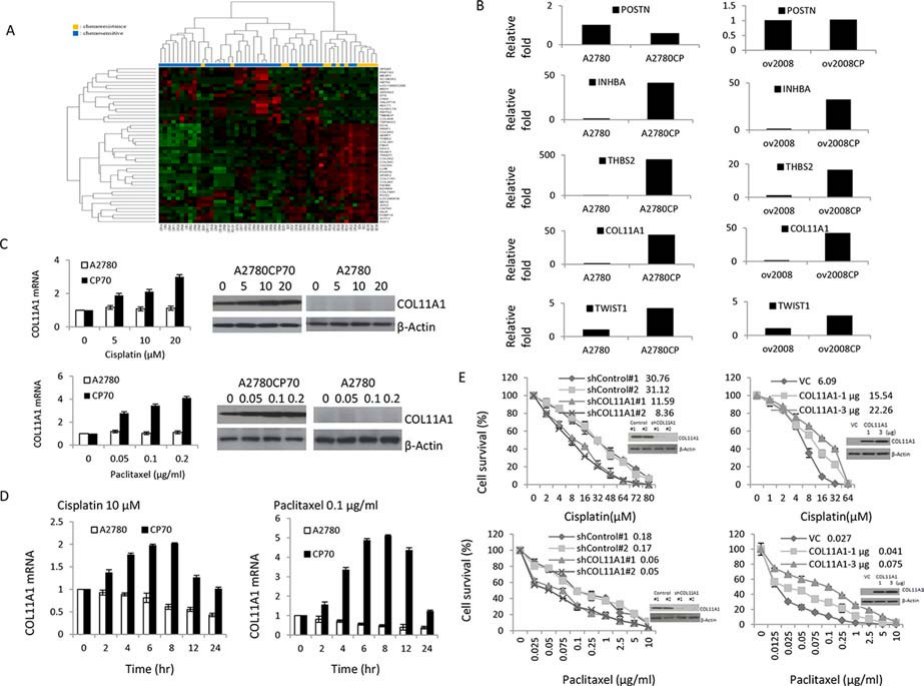
COL11A1 is involved in the regulation of cisplatin and paclitaxel responsiveness in chemoresistant ovarian cancer cells **A.** Identification of elevated COL11A1 levels in chemoresistant ovarian cancer using expression profiling. Hierarchical clustering of 60 samples using the 47 genes that were differentially expressed between patients with a good response to chemotherapy and those with a poor response. **B.** *POSTN, INHBA, THBS2, COL11A1*, and *Twist1* mRNA levels were evaluated by real-time RT-PCR in A2780, A2780CP70, ov2008, and ov2008CP20 ovarian cancer cells. **C.** COL11A1 expression was evaluated by real-time RT-PCR and western blotting in A2780 and A2780CP70 cells treated with different concentrations of cisplatin or paclitaxel. β-Actin was used as a protein loading control. All experiments were performed in triplicate. **D.** COL11A1 expression was evaluated by real-time RT-PCR in A2780 and A2780CP70 cells treated with cisplatin or paclitaxel for the indicated times. All experiments were performed in triplicate. **E.** A2780CP70 cells were transiently transfected with one of two COL11A1 small interfering RNAs (siCOL11A1 #1 and #2), and A2780 cells were transiently transfected with a *COL11A1* cDNA plasmid. After 48 h, cells were seeded into a 96-well plate and treated with various concentrations of cisplatin or paclitaxel for 72 h, and then cell sensitivity to anticancer drugs was measured by the MTT assay. Luciferase activity was measured and normalized to *Renilla* luciferase activity. COL11A1 protein expression in whole cell lysates was evaluated by western blotting. β-Actin was used as a protein loading control. All experiments were performed in triplicate.

Microarray analysis of tissue samples from 60 EOC patients showed that 47 genes (30 upregulated and 17 downregulated) were differentially expressed between chemoresistant and chemosensitive tumours (Table 1). An unsupervised heat map of these 47 genes with hierarchical clustering of the rows and columns is shown in Fig. 1A.

The five most differentially expressed genes (*POSTN, COL11A1, INHBA, TWIST1*, and *THBS2*) were selected and real-time RT-polymerase chain reaction (PCR) data both confirmed the reliability of our expression data and identified *COL11A1* as the most highly elevated transcript linked to chemoresistance, with an 8-fold increase compared to that of the control. For four of the five genes, we found a significant difference in gene expression levels between the chemosensitive and chemoresistant cell line pairs (Fig. 1B). Therefore, we selected *COL11A1* for further experimental analysis.

### COL11A1 is involved in the regulation of responsiveness to cisplatin and paclitaxel in chemoresistant ovarian cancer cells

Because *COL11A1* mRNA levels were higher in cisplatin-resistant A2780CP70 cells than in cisplatin-naive A2780 cells (Fig. 1B), we hypothesized that COL11A1 is preferentially activated by anticancer drugs, especially in cells with pre-existing chemoresistance. To test this, A2780 and A2780CP70 cells were treated with cisplatin, paclitaxel, gemcitabine, or pegylated liposomal doxorubicin (LipoDox^®^). Our data show that in A2780CP70 cells, COL11A1 was induced by both cisplatin and paclitaxel in a dose- (Fig. 1C) and time-dependent (Fig. 1D) manner. In contrast, COL11A1 expression was not induced by gemcitabine or LipoDox^®^ treatment (Supplementary Fig.1A). In addition, COL11A1 expression levels in A2780 cells were basically not activated by these four drugs (Fig. 1C, 1D, and Supplementary Fig. 1A).

To examine whether COL11A1 confers resistance to anticancer drugs, a small interfering RNA specific for the *COL11A1* gene (shCOL11A1) was introduced into A2780CP70 cells, and a *COL11A1* cDNA plasmid was introduced into COL11A1-low expressing A2780 cells to induce its overexpression. As expected, COL11A1 expression decreased in COL11A1-knockdown A2780CP70 cells and increased in COL11A1-overexpressing A2780 cells. The half-maximal inhibitory concentration (IC50) of cisplatin and paclitaxel was lower in COL11A1-knockdown A2780CP70 cells than in shcontrol cells. Conversely, the sensitivity of COL11A1-overexpressing A2780 cells to cisplatin and paclitaxel was decreased compared to that of vector control cells (VC) (Fig. 1E). The gemcitabine and LipoDox^®^ IC50 values were not changed by COL11A1 knockdown (Supplementary Fig. 1B). Collectively, these data demonstrate that COL11A1 is involved in the regulation of cisplatin and paclitaxel responsiveness in chemoresistant A2780CP70 cells but not in chemosensitive A2780 cells.

### The Akt/c/EBPβ signalling pathway is involved in cisplatin- and paclitaxel-induced COL11A1 upregulation in chemoresistant ovarian cancer cells

**Figure 2:**
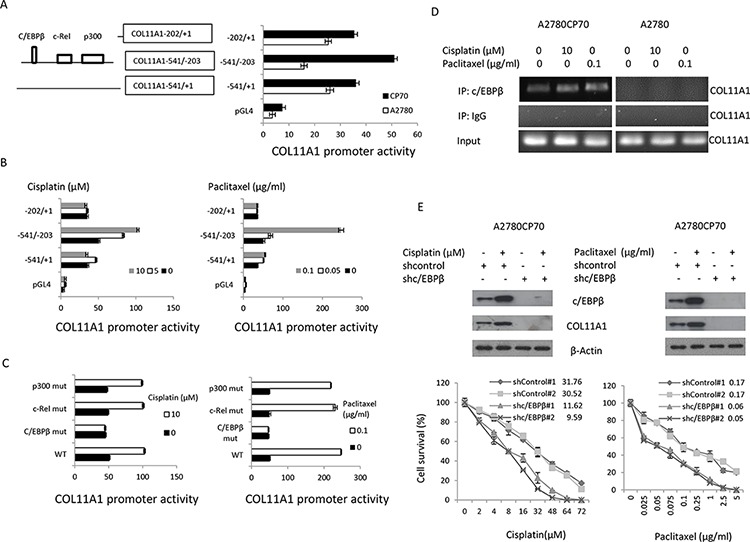
The c/EBPβ binding site in the COL11A1 promoter is the major determinant of COL11A1 activation by anticancer drugs in chemoresistant ovarian cancer cells **A.** Characterization of *COL11A1* promoter activity with a luciferase reporter assay. The *COL11A1* promoter deletion constructs and the luciferase reporter activity in A2780 and A2780CP70 cells transiently transfected with pGL4 vectors containing these deletion constructs are shown. **B.** A2780CP70 cells transfected with the indicated *COL11A1* promoter constructs were treated with various concentrations of cisplatin or paclitaxel for 8 h, and then luciferase activity was measured and normalized to *Renilla* luciferase activity. All experiments were performed in triplicate. **C.** A series of deletion mutants of the *COL11A1* −541/−203 promoter were introduced into A2780CP70 cells that were treated with cisplatin or paclitaxel. Luciferase activity was measured and normalized to *Renilla* luciferase activity. All experiments were performed in triplicate. **D.** A ChIP assay was performed to evaluate c/EBPβ binding to the *COL11A1* promoter after treatment of A2780CP70 and A2780 cells for 8 h with different concentrations of cisplatin or paclitaxel. **E.** A2780CP70 cells were transiently transfected with shc/EBPβ for 48 h and then treated with different concentrations of cisplatin or paclitaxel. After 8 h, c/EBPβ and COL11A1 protein expression was evaluated by western blotting. β-Actin was used as a protein loading control. Cell sensitivity to anticancer drugs was measured by the MTT assay. All experiments were performed in triplicate.

To further explore the mechanism by which the anticancer drugs cisplatin and paclitaxel increase *COL11A1* transcription, a fragment spanning positions −541 to −1 relative to the *COL11A1* transcription start site was amplified by PCR, sequenced, and cloned into a luciferase reporter plasmid. Then, a series of *COL11A1* promoter constructs containing various deletions (as shown in Fig. 2A) were constructed and transiently transfected individually into A2780 and A2780CP70 cells. These cells were then treated with anticancer drugs for 8 h. The data showed that *COL11A1* promoter activity was higher in cisplatin-resistant A2780CP70 cells than in cisplatin-naive A2780 cells (Fig. 2A). In addition, the luciferase activity of the transfectants containing the COL11A1−541/−203 promoter fragment was significantly enhanced by both cisplatin and paclitaxel in a dose-dependent manner. The luciferase activity of the COL11A1–541/+1 promoter fragment was weakly induced by both cisplatin and paclitaxel treatment, while that of the COL11A1–202/+1 promoter fragment was relatively unaltered by the treatments (Fig. 2B). In contrast, the activity of the *COL11A1* promoter was not stimulated by either gemcitabine or LipoDox^®^ treatment (Supplementary Fig. 1C). These results indicate that the region between−541 and−203 of the *COL11A1* promoter is important for transcriptional regulation by cisplatin and paclitaxel.

**Figure 3:**
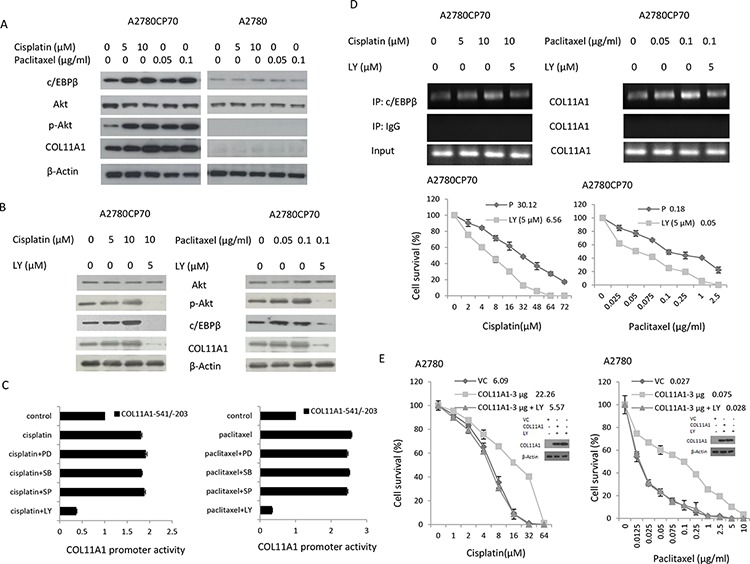
COL11A1 expression is increased by anticancer drugs via the Akt signalling pathway through c/EBPβ activation in chemoresistant ovarian cancer cells **A.** c/EBPβ, Akt, p-Akt, and COL11A1 protein expression in A2780CP70 and A2780 cells was evaluated by western blotting after treatment with different concentrations of cisplatin or paclitaxel for 8 h. **B.** Akt, p-Akt, c/EBPβ, and COL11A1 protein expression in A2780CP70 cells was evaluated after treatment with cisplatin or paclitaxel for 8 h in the presence or absence of PI3K inhibitor (LY). β-Actin was used as a protein loading control. **C.** A2780CP70 cells transfected with the *COL11A1* −541/−203 promoter were treated with cisplatin or paclitaxel for 8 h with or without various signalling pathway inhibitors. Luciferase activity was measured and normalized to *Renilla* luciferase activity. All experiments were performed in triplicate. **D.** A ChIP assay was performed to evaluate c/EBPβ binding to the *COL11A1* promoter in A2780CP70 cells after treatment with cisplatin or paclitaxel for 8 h in the presence or absence of PI3K inhibitor (LY). Sensitivity to anticancer drugs was measured by the MTT assay. All experiments were performed in triplicate. **E.** A2780 cells were transiently transfected with a COL11A1 expression plasmid for 48 h and then treated with cisplatin or paclitaxel in the presence or absence of a PI3K inhibitor (LY). After 8 h, sensitivity to anticancer drugs was measured by the MTT assay. All experiments were performed in triplicate.

TRANSFAC predicted putative p300, c-Rel, and c/EBPβ binding sites in the region between −541 and −203 of the *COL11A1* promoter (Fig. 2A). To determine which transcription factor is involved, *COL11A1* promoters with mutations in the p300, c-Rel, and c/EBPβ binding sites were constructed by site-directed mutagenesis. The activity of the *COL11A1* promoter reporter plasmids containing p300 or c-Rel binding site mutations was enhanced by cisplatin and paclitaxel, while the activity of reporter plasmids carrying a c/EBPβ binding site mutation was nearly unchanged (Fig. 2C). ChIP analysis showed that c/EBPβ binding to the *COL11A1* promoter region was stimulated by cisplatin and paclitaxel in A2780CP70 cells but not in A2780 cells (Fig. 2D). Silencing of EBPβ by shc/EBPβ suppressed the increase in COL11A1 expression induced by cisplatin and paclitaxel and increased sensitivity to cisplatin and paclitaxel in A2780CP70 cells (Fig. 2E). These results indicated that the c/EBPβ binding site in the *COL11A1* promoter is the major determinant of COL11A1 activation by anticancer drugs in chemoresistant ovarian cancer cells.

c/EBPβ expression has been shown to be increased by activation of the Akt pathway [14]. To examine the involvement of Akt signalling, A2780CP70 cells were treated with cisplatin or paclitaxel plus the phosphatidylinositol 3-kinase (PI3K) inhibitor LY294002. We found that the expression levels of p-Akt, c/EBPβ, and COL11A1 were enhanced by cisplatin and paclitaxel (Fig.3A) and that this elevated expression was inhibited by LY294002 (Fig.3B). Then, the COL11A1–541/–203 promoter constructs were transiently transfected into A2780CP70 cells, which were then treated with cisplatin or paclitaxel along with various signalling pathway inhibitors. As shown in Fig. 3C, the increase in luciferase activity induced by cisplatin or paclitaxel in the COL11A1–541/–203 promoter fragment-transfected cells was not decreased by inhibitors of extracellular signal-regulated kinase (ERK; PD98059), p38 (SB203580), c-Jun NH_2_-terminal kinase (JNK; SP600125), or TGF-β1 (Supplementary Fig. 2); however, the increase was almost completely inhibited by the PI3K inhibitor LY294002. ChIP analysis showed that c/EBPβ binding to the *COL11A1* promoter region was stimulated by cisplatin and paclitaxel and inhibited by LY294002 (Fig. 3D). Sensitivity of COL11A1-overexpressing A2780 cells to cisplatin and paclitaxel also increased after treatment with LY294002 (Fig. 3E). These results indicated that the Akt signalling pathway is involved in COL11A1-induced chemoresistance of ovarian cancer cells, which occurs through activation of c/EBPβ.

### COL11A1 increases the levels of phosphorylated Akt in chemoresistant ovarian cancer cells through stabilization of PDK1

**Figure 4:**
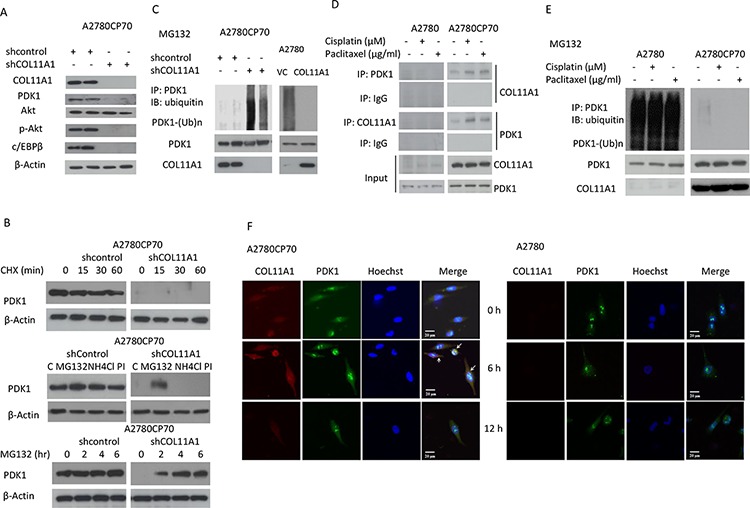
PDK1 protein is stabilized by COL11A1 through the increased binding of PDK1 to COL11A1 in chemoresistant ovarian cancer cells **A.** COL11A1, PDK1, Akt, p-Akt, and c/EBPβ protein expression in A2780CP70 cells transfected with shCOL11A1 was evaluated by western blotting. β-Actin was used as a protein loading control. **B.** Upper panel: A2780CP70 cells transfected with shCOL11A1 were incubated with cycloheximide (CHX: 20 mg/mL) for the indicated times and analysed by western blotting. β-Actin was used as a protein loading control. Middle panel: A2780CP70 cells transfected with shCOL11A1 were incubated with MG132 (20 μM), NH_4_Cl (10 μM), or protease inhibitors (1 mg/mL) for 6 h and analysed by western blotting. β-Actin was used as a protein loading control. Lower panel: A2780CP70 cells transfected with shCOL11A1 were treated with MG132 (10 μM) for the indicated times and analysed by western blotting. β-Actin was used as a protein loading control. **C.** A2780CP70 cells transfected with shCOL11A1 were treated with MG132 for 6 h, and then the cell lysates were immunoprecipitated with anti-PDK1 antibodies. The immunoprecipitates (IPs) were analysed by western blotting (IB) using an anti-ubiquitin antibody. **D.** A2780CP70 and A2780 cells were treated with cisplatin (10 μM) or paclitaxel (0.05 μg/mL) for 6 h, and then the cell lysates were immunoprecipitated with anti-PDK1, anti-COL11A1, and anti-IgG antibodies, and the IPs were analysed by IB. **E.** A2780 and A2780CP70 cells were treated with cisplatin (10 μM) or paclitaxel (0.05 μg/mL) and MG132 for 6 h, and then the cell lysates were immunoprecipitated with anti-PDK1 antibodies. The IPs were analysed by IB using an anti-ubiquitin antibody. **F.** Confocal microscopy images of COL11A1 and PDK1 immunofluorescence in A2780 and A2780CP70 cells treated with cisplatin (10 μM) for the indicated times. Scale bar, 20 μm.

As mentioned earlier, p-Akt protein levels varied in accordance with changes in COL11A1 expression induced by anticancer drugs or COL11A1 silencing (Fig. 2E). Phosphoinositide-dependent kinase-1 (PDK1) has been identified as the activator of *Akt* [15–17], and the kinase domain of PDK1 is ubiquitinated [18]. We then examined the expression of PDK1, p-Akt, and c/EBPβ and found that their expression was inhibited in shCOL11A1 cells (Fig. 4A). Therefore, we hypothesized that PDK1 protein stability could be enhanced by COL11A1. A2780CP70 cells transfected with shCOL11A1 or shcontrol were treated with cycloheximide, an inhibitor of protein synthesis. Western blotting showed that the half-life of PDK1 protein was significantly reduced in shCOL11A1-transfected cells compared to that in shcontrol-transfected cells (Fig. 4B, upper panel). Because PDK1 protein stability is reduced in shCOL11A1 cells, we next investigated the major route of PDK1 degradation, i.e., the proteasome, lysosome, or protease protein-degradation pathway. Fig. 4B shows that more PDK1 protein was present following treatment with a specific inhibitor of the proteasome pathway (MG132), whereas no significant change in PDK1 protein levels was observed after treatment with a lysosome inhibitor (NH_4_Cl) or protease inhibitor (PI). In addition, after MG132 treatment, PDK1 was degraded faster in shCOL11A1 cells than in shcontrol cells (Fig. 4B, lower panel). Therefore, the proteasome pathway is the major pathway involved in COL11A1 silencing-induced degradation of PDK1. Notably, after MG132 treatment, the pattern of PDK1 ubiquitination in A2780 cells was more extensive than that in A2780CP70 cells, and it could be rescued by exogenous overexpression of COL11A1. Conversely, COL11A1 silencing facilitated ubiquitination of PDK1 in A2780CP70 cells (Fig. 4C). Immunoprecipitation assays showed that the binding between COL11A1 and PDK1 was enhanced by treatment with anticancer drugs (Fig. 4D), which was in agreement with the suppressed PDK1 degradation observed in A2780CP70 cells (Fig. 4E). These changes were not observed in A2780 cells (Figs. 4D and 4E). The relationship between COL11A1 and PDK1 was further examined by immunofluorescence in A2780 and A2780CP70 cells treated with cisplatin (Fig. 4F). In A2780 cells, COL11A1 staining was minimal or absent, and the intensity and nuclear localization of PDK1 were not increased by cisplatin treatment. In contrast, A2780CP70 cells contained both cytoplasmic and nuclear clusters of COL11A1 and PDK1 immunoreactivity. Cisplatin treatment enhanced COL11A1 and PDK1 staining at 6 h. COL11A1-PDK1 colocalization was primarily detected in the perinuclear region (Fig. 4F). Collectively, these results indicate that PDK1 stabilization in chemoresistant ovarian cancer cells could be increased by COL11A1.

**Figure 5:**
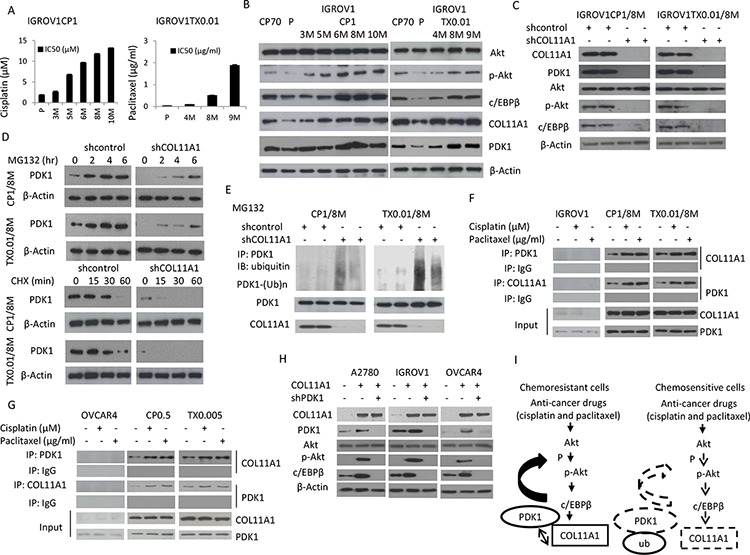
COL11A1 increases phosphorylated Akt via stabilization of PDK1 protein in chemoresistant ovarian cancer cells **A.** Sensitivity to cisplatin or paclitaxel in IGROV1-derived cisplatin- (CP1) or paclitaxel- (TX0.01) resistant cells was evaluated by the MTT assay. **B.** Western blotting was used to evaluate Akt, p-Akt, c/EBPβ, COL11A1, and PDK1 protein expression in IGROV1CP1 and IGROV1TX0.01 cells. β-Actin was used as a protein loading control. **C.** COL11A1, PDK1, Akt, p-Akt, and c/EBPβ protein expression in IGROV1CP1/8M and IGROV1TX0.01/8M cells transfected with shCOL11A1 was evaluated by western blotting. β-Actin was used as a protein loading control. **D.** Upper panel: IGROV1CP1/8M and IGROV1TX0.01/8M cells transfected with shCOL11A1 were treated with MG132 (10 μM) for the indicated times and analysed by western blotting. β-Actin was used as a protein loading control. Lower panel: IGROV1CP1/8M and IGROV1TX0.01/8M cells transfected with shCOL11A1 were incubated with cycloheximide (CHX: 20 mg/mL) for the indicated times and analysed by western blotting. β-Actin was used as a protein loading control. **E.** IGROV1CP1/8M and IGROV1TX0.01/8M cells transfected with shCOL11A1 were treated with MG132 for 6 h, and then the cell lysates were immunoprecipitated with anti-PDK1 antibodies. The resulting IPs were analysed by IB, using an anti-ubiquitin antibody. **F.** IGROV1, IGROV1CP1/8M, and IGROV1TX0.01/8M cells were treated with cisplatin or paclitaxel, and then the cell lysates were immunoprecipitated with anti-PDK1, anti-COL11A1, and anti-IgG antibodies, and the resulting IPs were analysed by IB. **G.** OVCAR4, OVCAR4CP0.5, and OVCAR4TX0.005 cells were treated with cisplatin or paclitaxel, and then the cell lysates were immunoprecipitated with anti-PDK1, anti-COL11A1, and anti-IgG antibodies, and the resulting IPs were analysed by IB. **H.** A2780, IGROV1, and OVCAR4 cells were co-transfected with COL11A1 and shPDK1 plasmids for 48 h, and then COL11A1, PDK1, p-PDK1, Akt, p-Akt, and c/EBPβ protein expression was evaluated by western blotting. β-Actin was used as a protein loading control. **I.** A hypothetical model illustrating the role of COL11A1 in the control of chemosensitivity in ovarian cancer cells.

In order to elucidate the mechanism underlying the emergence of chemoresistance in ovarian cancer cells, we established cisplatin- and paclitaxel-resistant strains by chronic exposure of IGROV1, ES2, and OVCAR4 cells to increasing doses of cisplatin (up to 1 μM for IGROV1 and up to 0.5 μM for OVCAR4) and paclitaxel (up to 0.01 μg/mL for IGROV1 and up to 0.005 μg/mL for OVCAR4). Within 3 to 10 months, more than eight independent resistant IGROV1 clones were isolated at various times. The IC50 values for the CP1 (1 μM cisplatin) and TX0.01 (0.01 μg/mL paclitaxel) clones increased gradually as the duration of drug exposure increased (Fig. 5A). Notably, crossresistance to cisplatin and paclitaxel was observed (Supplementary Fig. 3). In the CP1 and TX0.01 cells, the levels of p-Akt, c/EBPβ, COL11A1, and PDK1 increased as the duration of drug exposure increased (Fig. 5B). Similar to the findings in A2780CP70 cells (Fig. 4), the expression levels of PDK-1, p-Akt, and C/EBPβ were decreased in shCOL11A1-transfected cells (Fig. 5C). This indicated that PDK1 proteins were degraded more rapidly in shCOL11A1 cells than in shcontrol cells (Fig. 5D). The results of a cycloheximide pulse-chase experiment indicated that the half-life of PDK1 protein was significantly lower in shCOL11A1 cells than in shcontrol cells (Fig. 5D). In addition, the pattern of PDK1 ubiquitination observed after MG132 treatment was also more extensive in shCOL11A1 cells than in shcontrol cells (Fig. 5E). Immunoprecipitation assays indicated that the binding between COL11A1 and PDK1 was elevated by treatment with anticancer drugs in cells that acquired chemoresistance, but not in the parenteral cells (Fig. 5F). Similar phenomena were observed in OVCAR4 cells that acquired chemoresistance (Supplementary Figures 4A and 4B; Fig. 5G). To determine whether PDK1 was required for the decrease in p-Akt levels induced by COL11A1 knockdown, PDK1 shRNA was introduced into various COL11A1-overexpresing cells to knockdown PDK1. The elevated p-Akt and C/EBPβ levels induced by *COL11A1* transfection were reduced by introduction of PDK1 shRNA (Fig. 5H). These results indicate that COL11A1 increases the levels of phosphorylated Akt by stabilizing PDK1 protein, which promotes chemoresistance.

### *COL11A1* mRNA levels are correlated with response to chemotherapy and clinical outcome in EOC patients

**Figure 6:**
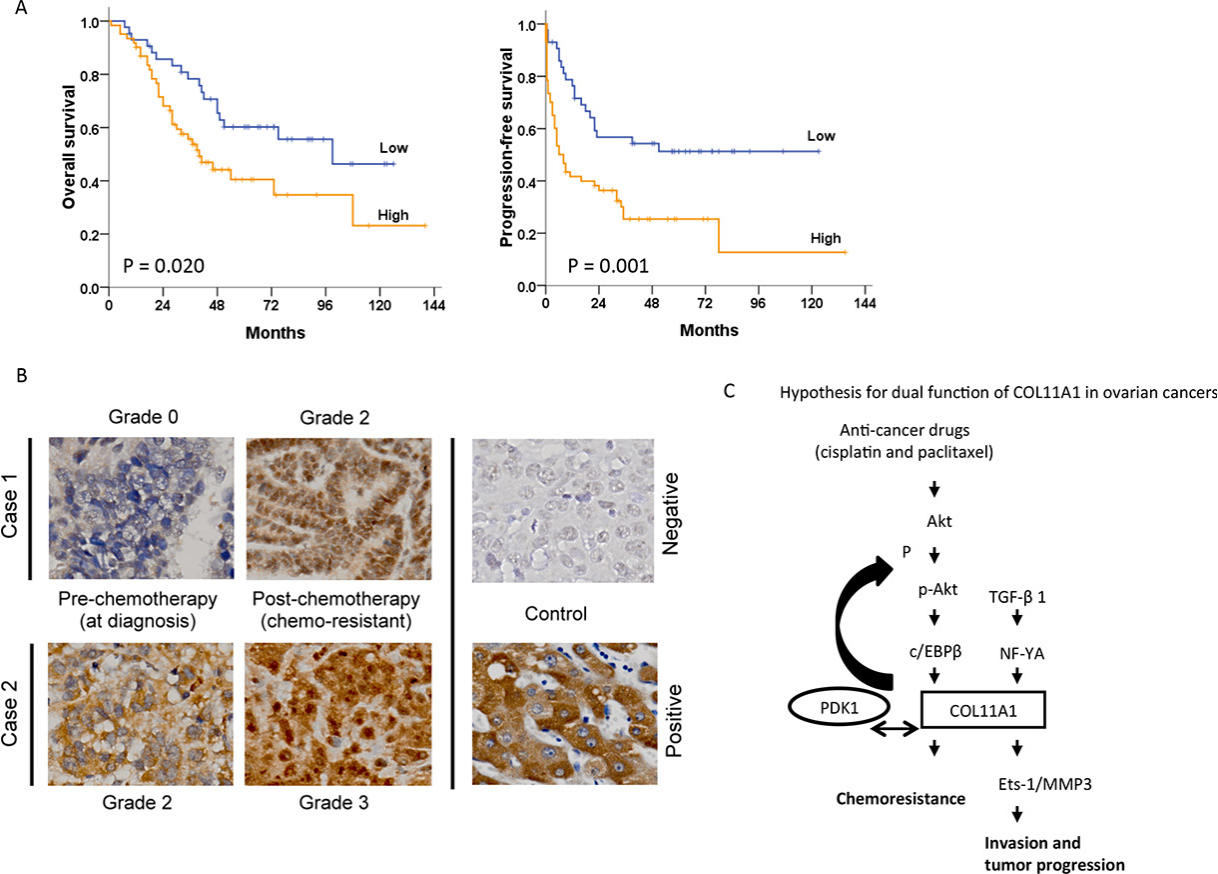
Association between COL11A1 expression and patient outcomes in ovarian cancer **A.** Kaplan-Meier curves are illustrated for 104 patients who underwent platinum-based chemotherapy. Patients with high COL11A1 mRNA levels in tumours had significantly poorer overall survival (*P* = 0.020) and progression-free survival (*P* = 0.001) than those with low COL11A1 levels. **B.** Increased COL11A1 protein expression in chemoresistant tumours. Cancer progression occurred after the 4^th^ and 5^th^ cycles of paclitaxel-carboplatin chemotherapy in cases 1 and 2, respectively. Both the intensity and the proportion of COL11A1-positive cancer cells was higher in chemoresistant cells than in chemo-naive cells (400 × magnification). **C.** A hypothetical model illustrating the dual role and regulation of COL11A1 in the control of cell aggressiveness and chemosensitivity in ovarian cancer cells.

The *COL11A1* mRNA expression and characteristics of 104 EOC patients are shown in Table 2. The median follow-up period was 44.5 months (range, 1–172 months). During follow-up, 64 patients (61.5%) experienced cancer progression, and 52 patients (50.0%) died. *COL11A1* mRNA expression levels in tumours were evaluated by real-time RT-PCR. A cut-off value of 1.8 was used to categorize the tumours into groups with high or low *COL11A1* mRNA levels. High *COL11A1* mRNA levels were significantly correlated with older age and advanced stage when compared to the levels in younger patients and at earlier stages (70.7% vs. 43.5%, *P* = 0.005 for age; 68.1% vs. 40.0%, *P* = 0.006 for International Federation of Gynaecology and Obstetrics [FIGO] stage). Additionally, high *COL11A1* expression levels were significantly associated with poor response to chemotherapy (*P* = 0.001), progression-free interval (PFI) ≤ 6 months (*P* < 0.001), and disease progression (*P* = 0.008). Patients with high *COL11A1* mRNA levels had significantly shorter overall survival (OS; *P* = 0.020) and progression-free survival (PFS; *P* = 0.001) compared to those with low *COL11A1* mRNA levels (Fig. 6A). Furthermore, IHC staining of COL11A1 protein in tumour tissue sections from two EOC patients with chemoresistant disease showed that the percentage of tumour cells stained positive for COL11A1 and the intensity of COL11A1 staining markedly increased after chemotherapy compared to those at initial diagnosis (Fig. 6B).

**Table 2:** Clinico-pathological characteristics and their associations with COL11A1 mRNA levels in epithelial ovarian cancer patients who experienced platinum-based chemotherapy (*n* = 104)

	*N* = 104	COL11A1 mRNA		*p*
		Low *n* (%)	High *n* (%)	
Age				0.005
≤ 52 years	46	26 (56.5)	20 (43.5)	
> 52 years	58	17 (29.3)	41 (70.7)	
Histology				0.029
Serous	52	16 (30.8)	36 (69.2)	
Non-serous	52	27 (51.9)	25 (48.1)	
Tumor grade				0.314
1 &2	33	16 (48.5)	17 (51.5)	
3	71	27 (38.0)	44 (62.0)	
Stage				0.006
Early	35	21 (60.0)	14 (40.0)	
Advanced	69	22 (31.9)	47 (68.1)	
Residual tumor size				0.046
< 1cm	82	38 (46.3)	44 (53.7)	
≥ 1cm	22	5 (22.7)	17 (77.3)	
Chemotherapy response				0.001
CR & PR	74	38 (51.4)	36 (48.6)	
SD & PD	30	5 (16.7)	25 (83.3)	
Progression-free interval				< 0.001
> 6 months	72	40 (55.6)	32 (44.4)	
≤ 6 months	32	3 (9.4)	29 (90.6)	
Progression				
No	40	23 (57.5)	17 (42.5)	0.008
Yes	64	20 (31.3)	44 (68.8)	

Data was analyzed by Chi-square test.

Non-serous type included clear cell (*n* = 21), endometrioid (*n* = 26) and mucinous (*n* = 5).

Early stage included stage I (*n* = 24) and stage II (*n* = 11).

Advanced stage included stage III (*n* = 58) and stage IV (*n* = 11).

Abbreviation: CR, complete response; PR, partial response; PD, progressive disease; SD, stable disease.

Serous type included only one grade 1 serous carcinomas with high COL11A1 level. This patient had cancer progression and platinum-resistant disease.

## DISCUSSION

We identified COL11A1 as a candidate biomarker of chemoresistance in ovarian cancer. The principal finding of our study is that EOC patients with COL11A1 overexpression had a higher rate of resistance to chemotherapy and shorter survival after first-line platinum-based regimens, indicating that elevated COL11A1 expression is an important chemoresistance marker of poor long-term survival. This finding was also demonstrated in chemoresistant cells derived from different subtypes of EOC. Resistant cells exhibited higher expression of COL11A1, c/EBPβ, Akt, and PDK1 than chemosensitive cells. We also found that drug sensitivity in chemosensitive cells could be reduced by overexpression of COL11A1 and that chemoresistance could be overcome by downregulation of COL11A1 or c/EBPβ. In a non-stressed state in chemosensitive cells, binding activity between COL11A1 and PDK1 was minimal and was unaltered by treatment with anticancer drugs, which in fact resulted in PDK1 ubiquitination and degradation. In chemoresistant cells, cisplatin enhanced c/EBPβ- and COL11A1-induced Akt-PDK1 binding, resulting in attenuation of PDK1 ubiquitination and degradation and protection of the cells from cytotoxic insults. Based on these findings, we propose that COL11A1 increases cancer chemosensitivity (Fig. 5I) and could serve as a therapeutic biomarker for EOC.

This study demonstrated that the c/EBPβ binding site in the *COL11A1* promoter (−541/−203) is necessary for activation of transcription by cisplatin and paclitaxel in chemoresistant ovarian cancer cells. The importance of c/EBPβ also stems from the finding that it functions as a mediator of cell survival and tumorigenesis [18, 19]. In addition, c/EBPβ expression is increased by activation of the Akt pathway [20]. Therefore, we identified Akt as a potential upstream regulator of c/EBPβ in chemoresistant ovarian cancer cells.

Overexpression and/or activation of the PI3K/Akt survival pathway have been shown to be associated with carcinogenesis in several tissue types [21–25]. One of the main downstream effectors of PI3K is the potent oncogenic serine/threonine kinase Akt, also known as protein kinase B (PKB) [26, 27]. Akt is activated by phosphorylation of Thr^308^ by the phosphoinositide-dependent kinase 1 (PDK1) [15, 16] and phosphorylation of Ser^473^ by the mammalian target of rapamycin complex 2 (mTORC2) [28]. Activated Akt functions to regulate several important molecular pathways, including those associated with cell survival, proliferation, and apoptosis. In the present study, we showed that the level of phosphorylated Akt was elevated in chemoresistant ovarian cancer cells and that treatment with an Akt inhibitor enhanced sensitivity to anticancer drugs. These findings are consistent with previous studies showing that Akt is a determinant of cisplatin sensitivity in chemoresistant ovarian cancer cells [29–35]. Here, we further showed that COL11A1 could increase phosphorylated Akt in chemoresistant ovarian cancer cells by stabilizing PDK1 protein. Further evidence of these observations is provided by the reduced PDK1 protein expression observed in COL11A1-knockdown cells, the marked increase in PDK1 protein in the presence of the 26S proteasome inhibitor MG132, and the proteasomal degradation of PDK1 observed in the presence of COL11A1 defects. We also observed that phosphorylated Akt levels in chemoresistant ovarian cancer cells were increased by COL11A1 via increased binding activity between PDK1 and COL11A1. Collectively, our results showed that overexpression of COL11A1 leads to chemoresistance, possibly by binding to PDK1-akt, and subsequently preventing PDK1 degradation. This binding may prevent cisplatin- and paclitaxel-induced PDK1 ubiquitination and degradation in these cells. To our knowledge, this study is the first report on the binding activity of COL11A1 and PDK1-akt in ovarian cancer cells under cisplatin and paclitaxel challenge. The results of this study suggest that COL11A1 may be an important determinant of chemoresistance that acts by sequestering PDK1 and preventing its ubiquitination and proteasomal degradation. Further studies using GST pull-down assays and/or X-ray crystallography analysis are needed to explore the binding activity of PDK1 and COL11A1.

Chemotherapy is an important therapeutic strategy for most patients with cancer. An understanding of the molecular mechanisms underlying chemoresistance in patients with aggressive cancers such as EOC might aid the design of new treatment strategies. Our results suggest that although cisplatin and paclitaxel exert their killing effects on ovarian cancer cells, they simultaneously induce the emergence of a subpopulation of cancer cells with activated Akt/c/EBPβ/COL11A1 signalling, which confers chemoresistance. In our study, ovarian cancer cells resistant to cisplatin or paclitaxel showed similar molecular changes, and cross-resistance between cisplatin and paclitaxel occurred in our cell models. This finding should alert clinicians to the possibility of cisplatin and paclitaxel cross-resistance and provide an explanation for the high recurrence rate following standard combination cisplatin and paclitaxel chemotherapy. However, cross-resistance was not observed between cisplatin/paclitaxel and gemcitabine/LipoDox^®^, and neither gemcitabine nor LipoDox^®^ treatment activated the Akt/c/EBPβ/COL11A1 signalling pathway. Gemcitabine and LipoDox^®^ are the most commonly used second-line therapy for EOC. This suggests that chemoresistance to anticancer drugs might be increased through distinct signalling pathways. Further research is needed to explore the mechanisms underlying cisplatin and paclitaxel cross-resistance and how chemoresistance to gemcitabine or LipoDox^®^ is decreased.

EOC is a highly heterogeneous disease that consists of four major histologic subtypes: serous, endometrioid, clear cell, and mucinous carcinoma. Our data show that COL11A1 is induced by cisplatin and paclitaxel through Akt/c/EBPβ in various ovarian cancer cell lines, including A2780CP70 (endometrioid), IGROV1 (mixed), ES2 (serous), and OVCAR4 (serous) [36, 37]. These findings suggest that regulation of COL11A1 transcription by cisplatin and paclitaxel via the Akt/c/EBPβ pathway is a relatively common phenomenon in EOC. One limitation of this study is the sample size for each of the EOC subtypes examined. Further investigation with a large-sample cohort is warranted to determine not only the COL11A1 alterations that occur during treatment but also the usefulness of COL11A1 for monitoring treatment responses compared with serial serum CA125 levels. Furthermore, more investigative efforts are needed to answer certain unresolved questions by determining the expression of COL11A1 and Akt-PDK1 in clinical tumour specimens from our cohorts.

We previously investigated the importance of COL11A1 in EOC. The results of our previous studies indicated that COL11A1 may promote cell aggressiveness via the TGF-β1/Ets-1/MMP3 axis and that the NF-YA binding site in the *COL11A1* promoter is the major determinant of TGF-β1-induced COL11A1 expression [7]. In the present study, we elucidated the mechanisms by which COL11A1 promotes cancer cell sensitivity to anticancer drugs, and we observed that, in ovarian cancer cells, chemoresistance developed via activation of the Akt/c/EBPβ pathway in concert with increased PDK1 degradation (Fig. 6C). The findings from our cell-based models were further supported by clinical evidence in EOC patients. Collectively, our findings highlight the importance of COL11A1 in EOC tumour progression and chemoresistance, and suggest that future therapies targeting COL11A1 or the Akt signalling pathway [38] might provide new opportunities for therapeutic intervention in chemoresistant EOC.

## MATERIALS AND METHODS

### Study population

For the microarray analysis, we obtained tissue from 60 patients with FIGO stage I–IV EOC who underwent comprehensive staging surgery or cytoreduction at our hospital between 2002 and 2010 [7]. Ovarian cancer specimens were intra-operatively collected following the standard protocol. We included a total of 104 EOC patients in the study. Review of medical records and pathologic slides for these patients provided clinical characteristics, pathologic diagnoses, and outcome information. The patients were followed up after treatment, and the date of the latest record retrieved was May 31, 2014. Both OS and PFS were calculated from the date of diagnosis, and PFI was measured to the date of last contact. EOC patients with PFIs ≤ or > 6 months were divided into “resistant” and “sensitive” to platinum-based chemotherapy groups, respectively. This study was approved by the institutional review board at our hospital (A-EX-102–015), and informed consent was obtained from all study subjects.

### Microarray data analysis for the identification of genes associated with chemoresponse in ovarian cancer

Gene expression arrays were generated from a subset of ovarian cancer patients who were described in our previous report [7]. The gene expression profiles of the samples were assessed using GeneChip^®^ Human Genome U133 2.0 Plus 2.0 arrays (Affymetrix, Santa Clara, CA, USA), and the raw data from the arrays have been made available to the public through Gene Expression Omnibus (GEO; accession number GSE44104). Samples from patients with stable disease (SD) or progressive disease (PD) after chemotherapy were defined as the chemoresistant group, and samples from patients with a complete response (CR) after chemotherapy were defined as the chemosensitive group. A gene was considered significantly differentially expressed if the fold change (up- or down-regulation) between the two groups was greater than 2-fold and the *t*-test *p*-value was less than 0.01. The fold change of a gene was calculated as the average expression in the chemoresistance samples divided by the average expression in the chemosensitive samples.

### Quantitative RT-PCR

Total RNA (5 μg) was used as the template in the cDNA synthesis reaction with random primers using Superscript III reverse transcriptase (Applied Biosystems). The resultant cDNA was used (at a 1:20 dilution) to detect the expression of endogenous *COL11A1* mRNA by qPCR. Accurate quantitation was achieved through the generation of standard curves by serially diluting a known amount of RNA from an *in vitro* transcription reaction and performing TaqMan qPCR on the series alongside the patient samples. Quantitative analysis of mRNA expression was performed using the Light Cycler^®^ 2.0 System (Roche Diagnostics GmbH). The primers and TaqMan probes used for the analyses were designed according to the manufacturer's software, Primer Express. The following primers were used: (a) COL11A1 forward primer 5′-TCGCATTGACCTTCCTCTTC-3′, reverse primer 5′-TCCCGTTGTTTTTGATATTC-3′, and probe 5′-6-FAM-CAGAGGAGCTGCTCCAGTTGATGT-TAMARA-3′; (b) INHBA forward primer 5′-TCATGCCAACTACTGCGAGG-3′, reverse primer 5′-ACAGTGAGGACCCGGACG-3′, and probe 5′-6- FAM-TGAGTGCCCGAGCCATATAGCAGGC-TAMARA-3′; and POSTN (HS00170815), TWIST1 (HS00361186), THBS2 (HS01568063), and GAPDH (HS99999905). No-reverse-transcription (no-RT) control reactions were performed using 100 ng of total RNA from each individual sample as a template to ensure that the amplification was not due to DNA contamination. No signal was detected in the no-RT controls. Target gene mRNA expression was assessed by real-time RT-PCR. The housekeeping gene *GAPDH* was used as the internal control for RNA quality. All of the quantitative analyses were performed in duplicate to assess the consistency of the results. The relative levels of the target gene, normalized to *GAPDH*, were expressed as ΔC_t_ = C_t_ (target)−C_t_ (GAPDH). The ratio of the number of copies of the target gene mRNA to the number of copies of *GAPDH* was then calculated as 2 ^−ΔCt^ × K (K = 10^6^, a constant).

### Cells and media

The human ovarian cancer cell lines A2780, A2780CP70, ov2008, ov2008CP20, IGROV1, and OVCAR4 were obtained from the American Type Culture Collection. The cisplatin- and paclitaxel-resistant strains of IGROV1 and OVCAR4 were developed in our laboratory. Cells were used between passages 5 and 20. Once resuscitated, the cell lines were routinely authenticated approximately every 6 months, and the cells were last tested in December 2014 through cell morphology monitoring, growth curve analysis, species verification by isoenzymology and karyotyping, identity verification using short tandem repeat profiling analysis, and contamination checks.

### Knockdown, expression vectors, and transfection

siRNAs directed against human COL11A1, c/EBPβ, and PDK1, and a non-targeting negative control siRNA (Control siRNA) were purchased from Santa Cruz Biotechnology. Another independent siRNA targeting COL11A1 and a scrambled control siRNA were obtained from OriGene (SR300918, OriGene). The COL11A1 cDNA (BC117697, GE Healthcare) was cloned with pCMV6-AC-GFP vector (PS100010, OriGene). Cancer cells were transfected with the siRNA or control siRNA using Lipofectamine 2000 (Invitrogen) for 48 h according to the manufacturer's protocol. The final concentration of the siRNAs was 10 nmol/L.

### Western blot analysis

Cells were washed in PBS, then lysed in lysis buffer containing 50 mM Tris-HCl (pH 7.5), 150 mM NaCl, 1 mM EDTA, 1 mM MgCl_2_, and 0.5% Triton X-100. Lysates were cleared by centrifugation at 13,000 × *g* for 20 min at 4°C and analysed by western blotting. Protein samples were separated by SDS-PAGE, transferred to a polyvinylidene difluoride membrane, and probed with the indicated antibodies. Antibody-bound proteins were detected by chemiluminescence.

### Antibodies and reagents

Anti-COL11A1 was obtained from Abcam. Anti-c/EBPβ, goat anti-mouse IgG-HRP and goat anti-rabbit IgG-HRP antibodies were purchased from Santa Cruz Biotechnology. Anti-Akt, anti-p-Akt, anti-PDK1, and anti-ubiquitin antibodies were obtained from Cell Signaling. Anti-β-actin antibody was obtained from Sigma. Cisplatin (Fresenius Kabi Oncology Ltd.), paclitaxel (Corden Pharma Latina S.P.A.), gemcitabine (Eli Lilly and Company), and pegylated liposomal doxorubicin (TTY Biopharm) were provided by the Cancer Center at National Cheng Kung University Hospital. ERK inhibitor (PD98059), p38/MAPK inhibitor (SB203580), JNK inhibitor (SP600125), and Akt inhibitor (LY294002) were obtained from InvivoGen. MG132 and cycloheximide (CHX) were obtained from Sigma-Aldrich.

### MTT cytotoxicity assay

The cells were cultured in a humidified incubator containing 95% air and 5% CO_2_ at 37°C in 96-well flat-bottomed microtiter plates. Before anticancer drug treatment, cells in exponential growth phase were transfected with the overexpression and knockdown plasmids for 48 h. After 72 h of incubation, the *in vitro* cytotoxic effects of these treatments were determined by the MTT assay (at 570 nm), and cell viability was expressed as a percentage of the viability of untreated control cells (% of control).

### Plasmid construction and site-directed mutagenesis

The *COL11A1* PCR product was cloned into the *Kpn*I and *Xho*I sites of the pGL4 vector. The resultant construct was confirmed by DNA sequencing. The *COL11A1* promoter deletion constructs COL11A1–541/+1, COL11A1–541/−203, and COL11A1–202/+1 were similarly generated using the COL11A1–541/+1 construct as a template. Site-directed mutagenesis was used to generate COL11A1–541/−203 promoter constructs containing the p300, c-Rel, and c/EBPβ mutant binding sites using complementary oligos (Supplementary Table 1).

### Luciferase reporter assay

Luciferase assays were conducted 48 h after transfection using a luciferase reporter assay system (Promega). Normalized luciferase activity is reported as luciferase activity/β-galactosidase activity.

### Chromatin immunoprecipitation (ChIP) assay

Native protein-DNA complexes were cross-linked by treatment with 1% formaldehyde for 15 min, and ChIP assays were performed as previously reported [39]. Briefly, equal aliquots of isolated chromatin were subjected to immunoprecipitation with anti-c/EBPβ and IgG monoclonal antibodies.

### Co-immunoprecipitation assay

Five to ten micrograms of anti-ubiquitin, anti-PDK1, and anti-COL11A1 antibody were added to an Eppendorf tube containing 500 μg of cold, pre-cleared protein lysate and incubated at 4°C overnight, and then 35 μL of washed, Protein A/G slurry (Santa Cruz) in pre-chilled lysis buffer was added the next day. The supernatant was carefully and completely removed, and the beads were washed 3–5 times with 500 μL of lysis buffer. After the last wash, the supernatant was aspirated, 50 μL of 1 × sample buffer was added to the bead pellet, and the sample was boiled for 10 min. After spinning at 13,000 × *g* for 5 min, the supernatant was collected for western blot analysis.

### Immunofluorescence

The cells were seeded and treated in chamber slides (Nalge Nunc) for the indicated times. The cells were then fixed in 3.7% paraformaldehyde, washed, and incubated in 0.05% Triton-X100 for 15 min. Next, the cells were blocked with SuperBlock solution (Thermo Scientific) for 1 h at room temperature. The cells were incubated with primary antibodies at 4°C overnight, washed with TBST, and then incubated with the appropriate secondary antibody (anti-goat Alexa-594 or anti-rabbit Alexa-488 [Molecular Probes]) and Hoechst 33342 dye for 1 h in the dark. Slides were then washed and fixed with GG1 gel (Sigma). Confocal images were obtained with an Olympus FV1000 microscope. The antibodies used for the immunofluorescence imaging were an anti-PDK1 antibody (Novus) and an anti-COL11A1 antibody (Santa Cruz).

### COL11A1 immunohistochemistry (IHC) of chemoresistant ovarian cancers

We included 2 chemoresistant patients who experienced secondary cytoreductive surgery to relieve bothersome symptoms when their disease progressed during paclitaxel-carboplatin doublet chemotherapy. Cancerous tissues from the ovarian site at diagnosis and peritoneal tumours at disease progression were fixed in formaldehyde, embedded in paraffin, and sectioned (4 μm thick). The sections were mounted on microscope slides, dried (16 h, 37°C), deparaffinized in xylene, rehydrated in graded alcohol, and rinsed with tap water. The sections were examined to confirm the histopathology after routine staining with hematoxylin and eosin. Briefly, IHC staining of COL11A1 protein was performed on deparaffinized tissue sections of formalin-fixed material after microwave-enhanced epitope retrieval based on the standard automated procedure (Ventana XT autostainer; Ventana Medical Systems, Tucson, AZ). The primary rabbit polyclonal anti-COL11A1 antibody (SC-68853, Santa Cruz Biotechnology) was used at a 1:20 dilution. In the negative control, the primary antibody was replaced with phosphate-buffered saline. Intensely COL11A1-positive normal hepatocytes were used as positive controls. The investigator (C-C Chen) was blinded to the clinical outcome of the specimens examined. The staining intensity was categorized as follows: negative = grade 0, weak = grade 1, moderate = grade 2, and strong = grade 3.

### Statistical analysis

Statistical analysis was performed using the Statistical Package for the Social Sciences software program (Version 11.0; SPSS Inc.). Frequency distributions between categorical variables were compared using Pearson chi-square test and Fisher's exact method. Survival was estimated using the Kaplan-Meier method and compared by log-rank tests.

## SUPPLEMENTARY FIGURES AND TABLE


