# Supplementary material for: COL11A1 confers chemoresistance on ovarian cancer cells through the activation of Akt/c/EBPβ pathway and PDK1 stabilization
Source: Oncotarget. 2015 Jun 10;6(27):23748–63. doi: 10.18632/oncotarget.4250 (PMC4695149; doi:10.18632/oncotarget.4250)
Supplement: Supplementary file 1 [file oncotarget-06-23748-s001.pdf]

SUPPLEMENTARY FIGURES AND TABLE

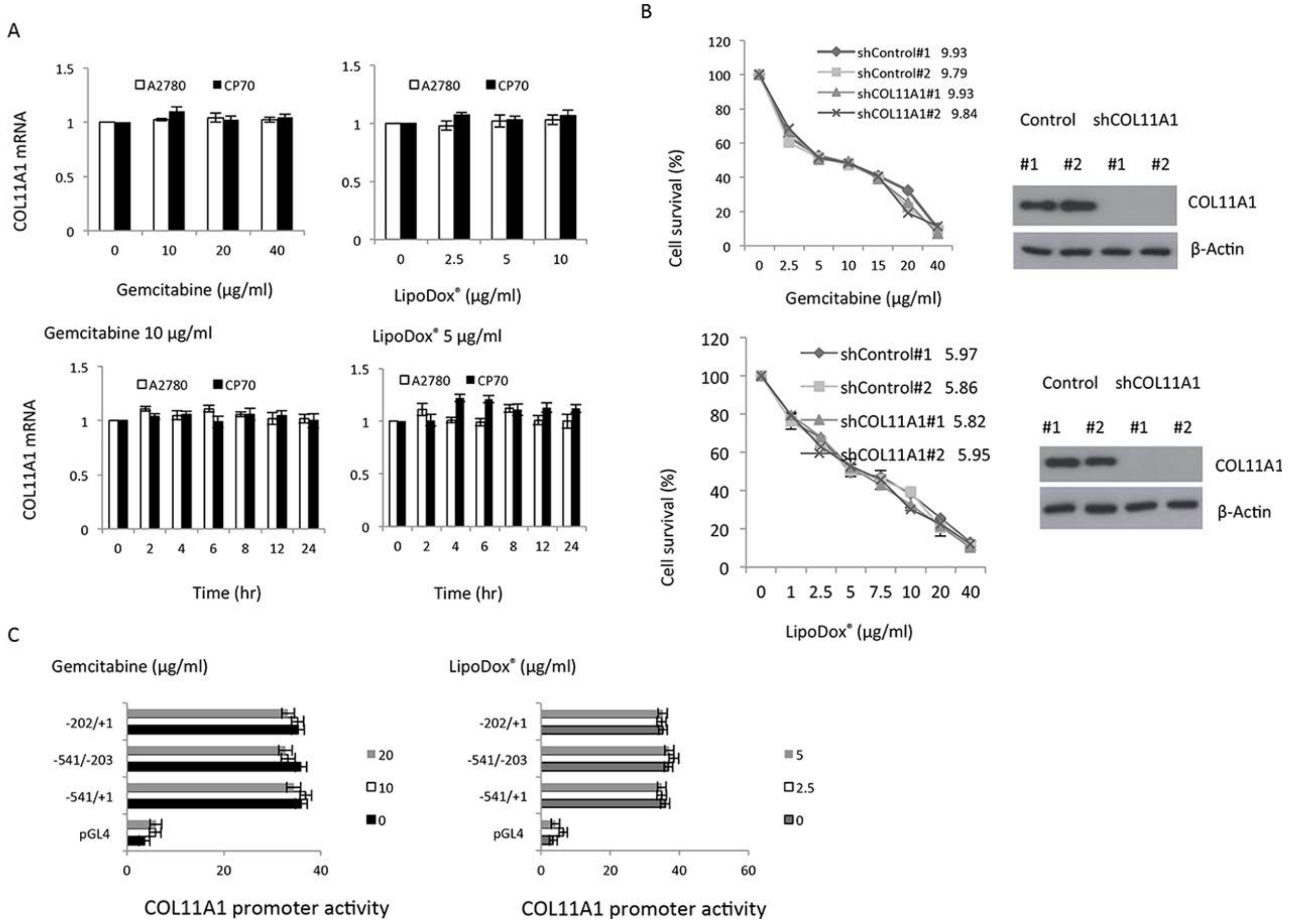

Supplementary Figure S1: COL11A1 expression was not affected by gemcitabine or LipoDox® treatment. A. Upper panel: COL11A1 expression was evaluated by real-time RT-PCR in A2780 and A2780CP70 cells treated with different concentrations of gemcitabine or pegylated liposomal doxorubicin (LipoDox®). All experiments were performed in triplicate. Lower panel: COL11A1 expression was evaluated by real-time RT-PCR in A2780 and A2780CP70 cells treated with gemcitabine or LipoDox® for the indicated times. All experiments were performed in triplicate. **B.** A2780CP70 cells were transiently transfected with each of two COL11A1 small interfering RNAs (siCOL11A1 #1 and #2). After 48 h, cells were seeded into a 96-well plate and treated with various concentrations of gemcitabine or LipoDox® for 72 h, and then cell sensitivity to these two drugs was measured by MTT assay. COL11A1 protein expression was evaluated by western blotting in whole cell lysates. β-Actin was used as a protein loading control. All experiments were performed in triplicate. **C.** The promoter activity of COL11A1 was not affected by gemcitabine or LipoDox® treatment.

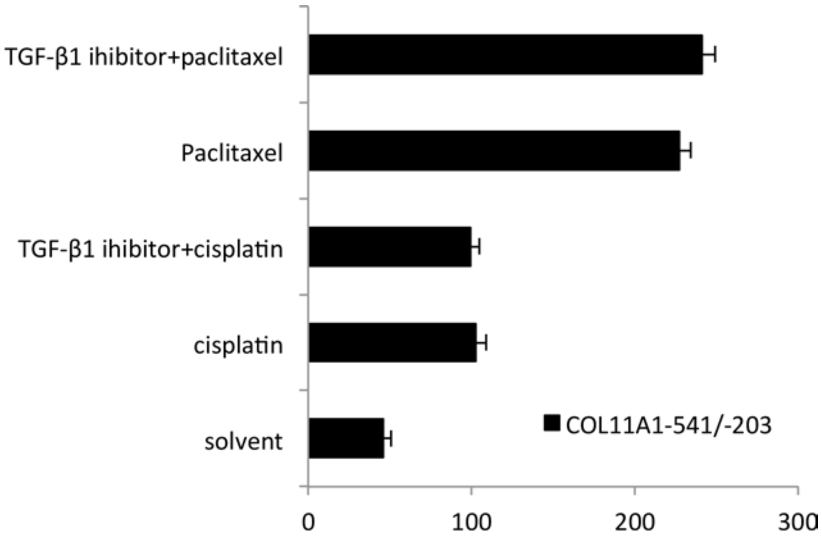

Supplementary Figure S2: The increase in luciferase activity induced by cisplatin or paclitaxel in the COL11A1–541/–203 promoter fragment-transfected cells was not affected by TGF-β1 inhibitor.

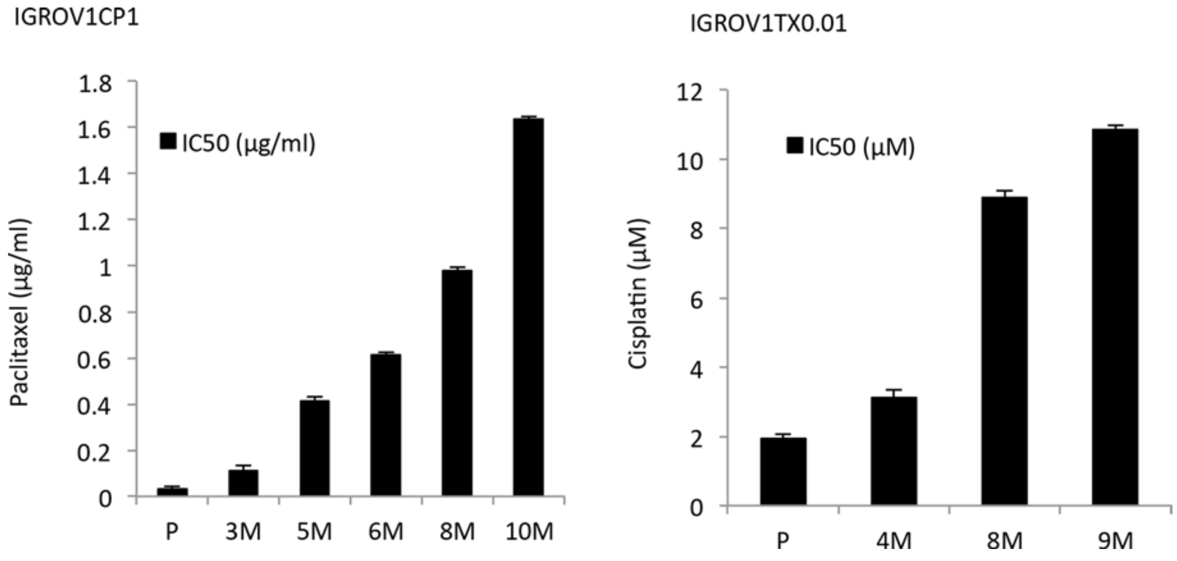

Supplementary Figure S3: Crossresistance to cisplatin and paclitaxel in IGROV1 cells that acquired chemoresistance.

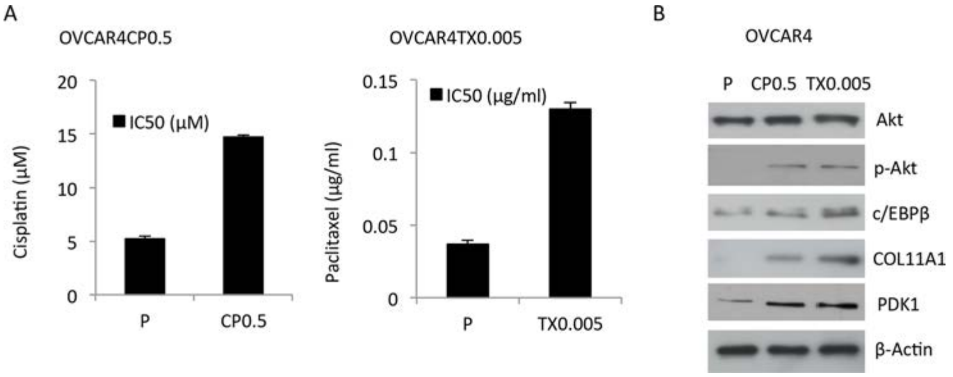

Supplementary Figure S4: Increased expression levels of PDK-1, p-Akt, COL11A1, and C/EBPβ in OVCAR4 cells that acquired chemoresistance. **A.** Cell sensitivity to cisplatin or paclitaxel was evaluated by MTT assay in OVCAR4-derived cisplatin- (CP0.5) or paclitaxel- (TX0.005) resistant cells. **B.** Western blotting was performed to evaluate the Akt, p-Akt, c/EBPβ and COL11A1 protein expressions in OVCAR4CP0.5 and OVCAR4TX0.005 cells. β-Actin was used as a protein loading control.

Supplementary Table S1. List of primer sequences used in COL11A1 promoter construction

Construct		Sequence
COL11A1–541/+1	Forward	GGTACCGCCATACGAAGCAGATT
	Reverse	CTCGAGGCCCCTAAAGGCTTCATG
COL11A1–202/+1	Forward	GGTACCCTCGGAGTCCTCATTCTTGG
	Reverse	CTCGAGGCCCCTAAAGGCTTCATG
COL11A1–541/−203	Forward	GGTACCGCCATACGAAGCAGATT
	Reverse	CTCGAGCAGGCCCAGCCCACCAA
COL11A1–541/−203 P300 mutant	Forward	CGCGTCCCCCGTAACTTTTTCCCATTTT
	Reverse	AAATGGGAAAAAGTTACAAAGGACGCG
COL11A1–541/−203c-Rel mutant	Forward	CTCCGTGTGCTCCAAAACTCTCCTGCAATT
	Reverse	AATTGCAGGAGAGTTTTGGAGCACACGGAG
COL11A1–541/−203c/EBPβ mutant	Forward	GCAGATTTTTGCGTGGGGATAAGGTTAC
	Reverse	GTAACCTTATCCCCACGCAAAAATCTGC
